# Pathogenic microRNAs Common to Brain and Retinal Degeneration; Recent Observations in Alzheimer’s Disease and Age-Related Macular Degeneration

**DOI:** 10.3389/fneur.2015.00232

**Published:** 2015-11-03

**Authors:** James M. Hill, Aileen I. Pogue, Walter J. Lukiw

**Affiliations:** ^1^Department of Ophthalmology, LSU Neuroscience Center, School of Medicine, Louisiana State University Health Sciences Center, New Orleans, LA, USA; ^2^Alchem Biotek, Toronto, ON, Canada; ^3^Neuroscience Center and Department of Neurology, LSU Neuroscience Center, School of Medicine, Louisiana State University Health Sciences Center, New Orleans, LA, USA

**Keywords:** complement factor H, disease heterogeneity, innate-immune and inflammatory response, miRNA-9, miRNA-34a, miRNA-125b, miRNA-146a, miRNA-155

MicroRNAs (miRNAs), ~22 nt single-stranded non-coding RNAs (ncRNAs) abundant in the human brain and retina, have emerged as significant post-transcriptional regulators of messenger RNA (mRNA) abundance and complexity in the human central nervous system (CNS) in aging, health, and disease. Of the 2050 different miRNAs in the human body so far identified, only about 25–30 are abundant in either the brain or the retina, underscoring the high selection pressure carried by RNA sequences located within these select ncRNAs (1–7). It is noteworthy to point out that: (i) that brain neocortex and retina share a common neuroectodermal origin; (ii) that brain and retina share a subfamily of specific miRNA species; and (iii) that the multilayered assemblies of both neural and retinal cells are targeted by pathogenic processes that drive progressive pro-inflammatory neurodegeneration (5–9). Indeed, pathologically up-regulated miRNAs common to both the prototypic age-related inflammatory degeneration of the brain in Alzheimer’s disease (AD) and of the retina in age-related macular degeneration (AMD) appear to be associated with deficits in the expression of messenger RNA (mRNA) and gene families involved in the innate-immune response, inflammation, neurotrophism, synaptogenesis, and amyloidogenesis (Figure 1). In this “Opinion” paper for the *Frontiers in Neurology Special Research Topic*, we will highlight some of the most recent work in this research area, with emphasis on a family of five up-regulated pro-inflammatory miRNAs – miRNA-9, miRNA-34a, miRNA-125b, miRNA-146a, and miRNA-155 – that are emerging as key mechanistic contributors to the AD and AMD process.

**Figure 1:**
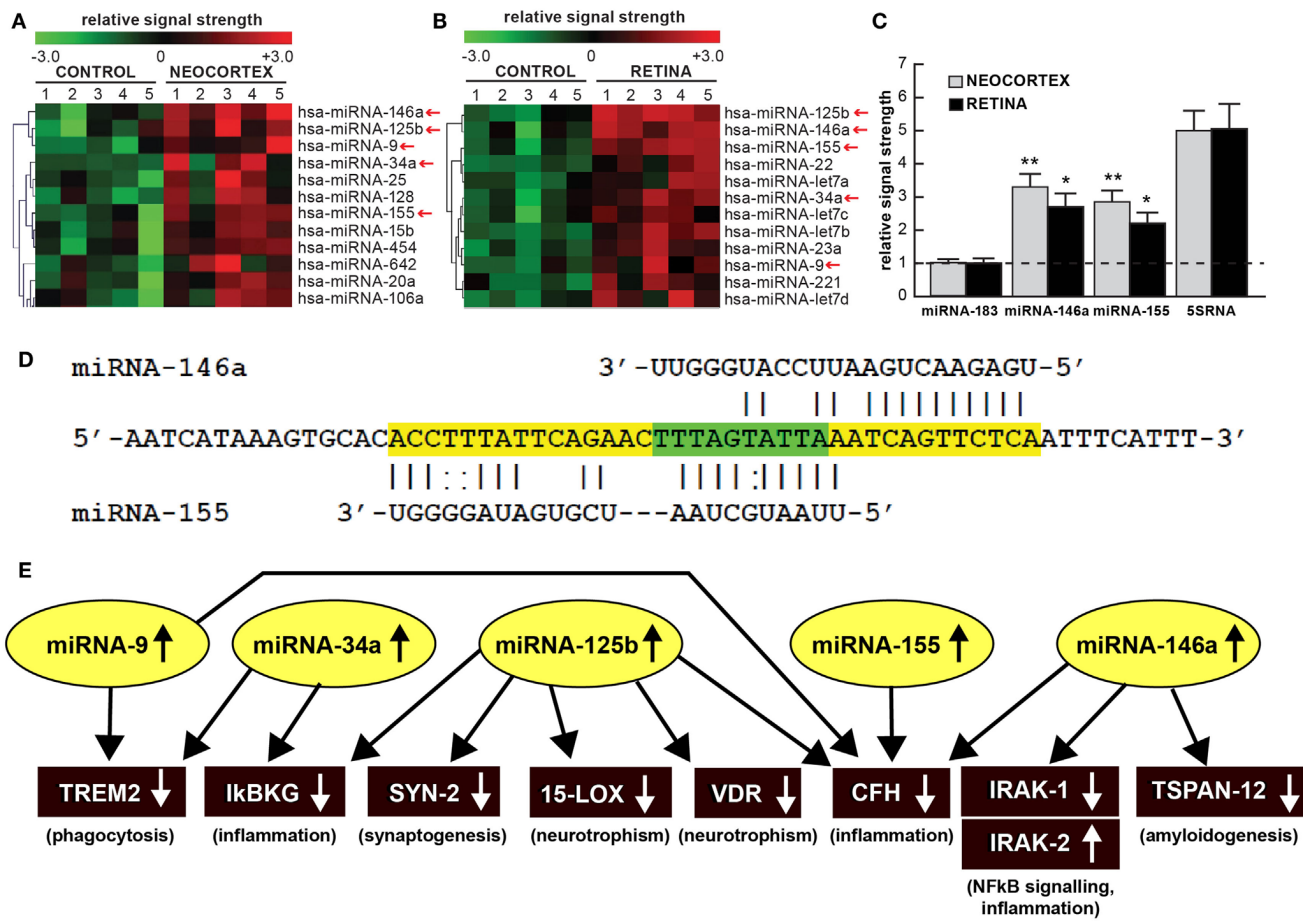
**(A)** Color-coded cluster analysis of significantly up-regulated microRNAs (miRNAs) in the neocortex of AD (*N* = 5) versus age-matched controls (*N* = 5) and **(B)** in the whole retina of AMD (*N* = 5) versus age-matched controls (*N* = 5); this small family of “pro-inflammatory” miRNAs consisting of miRNA-9, miRNA-34a, miRNA-125b, miRNA-146a, and miRNA-155 are often found to be up-regulated approximately twofold or more over controls (small red arrows); interestingly these miRNAs are inducible and under transcriptional control by NF-kB [Figure 1 adapted from Ref. (10–13)]; the relative expression levels for two sequence-related microRNAs, miRNA-146a and miRNA-155 the brain neocortex and retina are shown in **(C)** relative to levels for an unchanging brain and retinal control miRNA-183, which was set to 1.0 and marked by a dashed horizontal line; another abundant control signal is 5SRNA with a relative signal strength of ~5.0 (shown at ~1/20th of its actual abundance in the brain and retina; see text) (12, 13). In these samples, all control and AD neocortical samples were obtained from the superior temporal neocortex (Brodmann area A22); control and AMD retinal samples were obtained from whole retina; all control, AD and AMD samples had post-mortem intervals (PMI; death to brain freezing interval) of 2 h or less (2, 9, 10). Controls were age-matched to moderate-to-late stages of AD or AMD; increases in specific miRNAs increased as disease stage advanced [Ref. (11, 12, 14–17); data not shown]; further details on the pathology of these samples have been recently published (11–20). There were no significant differences in age, PMI, or RNA yield or quality between either the brain or the retinal tissues. Of the 12 different homo sapien micro-RNAs (hsa-miRNAs) shown, miRNA-146a and miRNA-155 exhibited the most consistent up-regulation compared with age-matched controls (*p < 0.05; **p < 0.01, ANOVA); **(D)** the 3′UTR of the mRNA of complement factor H (CFH); a major regulator of the innate-immune and inflammatory response, see text; [(21, 22)] is a prime example of brain and retinal gene expression regulation by multiple and common miRNAs – miRNA-146a and miRNA-155; **(D)** shows the complementarity map between miRNA-146a or miRNA-155 and part of the 232 nt CFH 3′UTR sequence. Overlapping miRNA-146a and miRNA-155 high-affinity binding sites in the CFH mRNA 3′UTR (each has an energy of association of less than −22 kcal/mol) that defines an exceptionally stable miRNA–mRNA interaction and a potentially common CFH mRNA 3′-UTR miRNA regulatory control region 5′-TTTAGTATTAA-3′ (overlaid in green; see text) (12, 13, 23); we cannot exclude the participation of other human brain- or retina-enriched miRNAs or other small ncRNAs which may additionally contribute to the neuropathological mechanisms of AD or AMD pathology; **(E)** taken together, these recent findings in part define a highly interactive network of NF-kB-sensitive, up-regulated miRNAs in diseased brain and retina that can explain much of the observed pathology associated with AD and AMD. The CNS-abundant, miRNA-125b is a central member of this up-regulated miRNA group that may be in part responsible for driving deficits in phagocytosis (triggering receptor expressed in microglial cells; TREM2), innate-immune signaling and chronic inflammation (IkBKG, CFH), impairments in neurotransmitter packaging and release (synapsin-2; SYN-2), and neurotrophism (15-lipoxygenase, vitamin D receptor; 15-LOX, VDR). Other NF-kB-sensitive up-regulated miRNAs (such as miRNA-146a) appear to be responsible for the observed deficits in NF-kB regulation (IRAK-1, IRAK-2) and/or amyloidogenesis (tertraspanin 12; TSPAN12); these up-regulated miRNAs and down-regulated mRNAs form a highly integrated, self-perpetuating pathogenic miRNA-mRNA signaling network due to chronic re-activation of NF-kB stimulation perhaps through the involvement of deficits in IkBKG signaling (10–13). Inhibition of the NF-kB initiator or individual blocking of the pathogenic induction of these five miRNAs may provide novel therapeutic benefit for the clinical management of AD or AMD, however what NF-kB or miRNA inhibition strategies and/or protocols, or whether they can be utilized either alone or in combination, remain open to investigation (17, 19, 20, 24, 25). Extensive recent data in human brain cells in primary culture have indicated that these approaches may neutralize this chronic, inducible, progressive pathogenic gene expression program to re-establish brain and retinal cell homeostasis, and ultimately be of novel pharmacological use in the clinical management of AD and/or AMD (19, 20, 26–30).

Homeostatic levels of specific miRNAs are natural indicators of neurological health of both the brain and retina (2–10, 31). Recently, multiple independent neurological research laboratories have provided evidence for the up-regulation of a small group of five inducible miRNAs in age-related diseases involving a progressive inflammatory degeneration. That these five miRNAs – miRNA-9, miRNA-34a, miRNA-125b, miRNA-146a, and miRNA-155 – are up-regulated in both AD and AMD underscores the concept that the brain and retina share common pathological signaling of a pre-existing subfamily of miRNAs that individually contribute to various aspects of neurodegenerative disease (5–12, 31–35). Accumulating evidence, including very recent research findings over the last 6 months indicate that each of these miRNAs share the following six features: (i) that they are basally expressed in control brain neocortex and retina (2–9); (ii) that *in vitro* they can be induced by a wide range of environmental- and inflammation-linked physiological stressors, including pro-inflammatory cytokines, amyloid beta (Aβ42) peptides, neurotoxic metal sulfates, and neurotropic viruses such as herpes simplex virus-1 (HSV-1) (12, 16, 17, 32–35); (iii) that this group of five pro-inflammatory miRNAs are over-expressed at least twofold in stressed brain or retinal cells and in AD or AMD affected tissues (14, 15, 32); (iv) that together, via down-regulation of multiple mRNA targets (and hence deficits in the expression of genes encoded by those mRNAs) they regulate various pathophysiological features characteristic of AD and AMD, including impairments in phagocytosis, synaptogenesis, neurotrophism, NF-kB signaling and stimulation of progressive inflammation and amyloidogenesis (Figure 1) (7, 12, 13, 23, 26–28, 36); (v) that all five of these pro-inflammatory miRNAs are under transcriptional control by NF-kB (chiefly the heterotypic p50/p65 dimer) in human primary neuronal-glial co-cultures, AD and AMD tissues (7, 11–13, 23, 26–28, 36, 37); and (vi) that both NF-kB inhibitors and anti-microRNAs (anti-miRs) effectively knock down their expression in human brain and retinal cell culture experiments, and may ultimately be of use therapeutically in the clinical management of AD or AMD (17, 18, 26–29).

Much of the recent research work emphasizing this commonality of the same miRNAs in basic pathological processes involving brain and retinal degeneration, as exemplified by miRNA profiling in AD, AMD, and transgenic AD or AMD (TgAD, TgAMD) models, has been summarized in Figure 1 (5–10, 12, 14, 17, 25, 31–35). First, when compared to the unchanging 22 nt miRNA-183 and the 120 nt 5S ribosomal RNA (5S rRNA; 5SRNA) control markers, the five member pro-inflammatory microRNAs miRNA-9, miRNA-34a, miRNA-125b, miRNA-146a, and miRNA-155 are found to be amongst the most consistently up-regulated miRNAs in both degenerating human brain neocortex (Figure 1A) and retina (Figure 1B). Of this group of five pro-inflammatory microRNAs, miRNA-146a and miRNA-155 are typically found to be increased ~2.5- to 3.3-fold over age-matched controls (Figure 1C). To add another layer of genetic complexity for post-transcriptional regulation, both miRNA-146a and miRNA-155 recognize an overlapping 3′ untranslated region (3′UTR) of the complement factor H (CFH) mRNA (highlighted in green; CFH loss-of-function mutations or CFH expression deficits are associated with both AD and AMD; see below; Figure 1D). Indeed, the up-regulation of these same five pro-inflammatory miRNAs (yellow ovals in Figure 1E) appear to form a highly interactive miRNA–mRNA network that can in part explain the down-regulation of specific brain and retinal genes (black rectangles) involved in phagocytosis, inflammation, synaptogenesis, neurotrophism, NF-kB signaling, and amyloidogenesis (Figure 1E; see also the legend to Figure 1 wherein the details of this highly interactive network are further described).

Alterations in innate-immune signaling are a consistent feature of both AD and AMD (4, 5, 9, 15). The highly soluble, hydrophilic 155-kDa glycoprotein CFH is one very illustrative example of an innate-immune repressor and complement control protein whose abundance and/or activity is significantly down-regulated in both AD and AMD [(9, 15, 21, 22, 35); see Figure 1D]. CFH (chr 1q32; also known as AC3bINA, adrenomedullin binding protein-1, AM binding protein-1 factor H, β1H globulin, H factor, and H factor-1) is an important member of the regulator of complement activation (RCA) group of proteins encoded within the RCA gene cluster and normally performs a systemic sentinel function against unscheduled or spontaneous immune system activation (9, 15). CFH mRNA abundance is down-regulated in AD and/or AMD by a miRNA-146a- and/or miRNA-155–CFH–3′UTR-based complementarity mechanism and/or by a Y402H loss-of-function mutation (15, 21, 22). Hence an insufficiency in a homeostatic amount of functioning CFH (as down-regulated by miRNA-146a and miRNA-155) may have the same end result as the loss-of-function Y402H mutation in CFH (21, 22). It is important to note that CFH mRNA and hence CFH gene expression appears to be down-regulated by at least two different miRNAs – miRNA-146a and/or miRNA-155 – and their differential recognition of overlapping binding sites in the human CFH mRNA 3′UTR may be dependent on yet-to-be-defined genetic factors and mechanisms characteristic of individual brain or retinal cells [Figure 1D; (9, 15, 21, 22, 35)].

In summary, it is our opinion that in miRNA research in human degenerative diseases including AD and AMD, several critical concerns have surfaced: (i) that brain and retinal miRNAs typically possess limited stabilities, however miRNA half-lives can be considerably extended via their sequestration into exosomes or the use of other protective strategies such as adsorption or tertiary folding into RNAse-resistant structures that may escape initial miRNA detection using traditional methods (17, 18, 23–25); (ii) that accurate quantification of miRNAs is technically feasible although it still remains challenging due to the small size of mature miRNA isoforms, adsorption to “inert” surfaces, high sequence homology amongst individual miRNAs, 5′ and 3′ end polymorphisms, spatial-temporal expression patterns and high dynamic range of miRNA expression (13, 17, 18, 24); (iii) that miRNA profiling in different AD or AMD studies suffers from a poor consensus regarding their abundance and complexity; the latter a very recently acknowledged concern in the field (4–7, 14, 17); and (iv) discrepancies of miRNA abundances in anatomical areas sampled, variations in patient drug history, the PMI of the AD and AMD patients and other factors. Together these constitute practical methodological challenges, especially in the realm of useful biomarkers and diagnostics for AD or AMD detection (3, 6, 17, 25, 34). Despite these recent concerns data has begun to filter through on the involvement of distinct miRNA families and miRNA–mRNA signaling networks linked to innate-immune system alterations, inflammatory, neurotrophic, and amyloidogenic consequences in AD and AMD. These have steadily yielded a deeper appreciation into the onset and propagation of complex miRNA–mRNA-modulated biological networks that directly underlie the pathogenesis of AD and AMD. Lastly, miRNAs are highly soluble and mobile, and are able to transverse plasma membranes either freely, adsorbed to carrier molecules or contained within exosomes (17, 19, 23, 25). That AD and AMD are both progressive “propagating” disease entities suggest a potential “spreading factor” role for selective miRNAs in the cognitive and visual circuitry, an evolving research area in which specific combinations of miRNAs may be playing hitherto unrecognized pathogenic roles.

## Conflict of Interest Statement

The authors declare that the research was conducted in the absence of any commercial or financial relationships that could be construed as a potential conflict of interest.

## Abbreviations

AMD, age-related macular degeneration; AD, Alzheimer’s disease; CFH, complement factor H; miRNA, microRNA
